# Lumbar puncture-administered resveratrol inhibits STAT3 activation, enhancing autophagy and apoptosis in orthotopic rat glioblastomas

**DOI:** 10.18632/oncotarget.12414

**Published:** 2016-10-03

**Authors:** Song Xue, Shu Xiao-Hong, Sha Lin, Bian Jie, Wang Li-Li, Gu Jia-Yao, Shi Shun, Li Pei-Nan, Wu Mo-Li, Wang Qian, Chen Xiao-Yan, Kong Qing-You, Zhang Peng, Li Hong, Liu Jia

**Affiliations:** ^1^ Liaoning Laboratory of Cancer Genomics and Department of Cell Biology, College of Basic Medical Sciences, Dalian Medical University, Dalian 116044, China; ^2^ Department of Radiology, Second Affiliated Hospital of Dalian Medical University, Dalian 116023, China; ^3^ Department of Orthopedics, Second Affiliated Hospital of Dalian Medical University, Dalian 116023, China

**Keywords:** resveratrol, orthotopic rat glioblastoma, lumbar puncture, STAT3 signaling, cell death

## Abstract

Trans-resveratrol suppresses glioblastoma growth *in vitro*, but its effects on intracranial glioblastomas remain untested. Resveratrol crosses the blood–brain barrier, and lumbar puncture (LP) greatly increases its bioavailability in rat brains; therefore, we investigated the effectiveness of LP-administered resveratrol on orthotopic rat glioblastomas. Twenty-four tumor-bearing rats were separated into two groups: Group 1 receiving 100 μl saline containing 0.3% DMSO and Group 2 receiving 100 μl resveratrol (300 μM). Treatments started 3 days after transplantation in 2-day intervals until death. Intracranial drug availabilities, tumor sizes, average life spans and the impacts on STAT3 signaling, apoptosis and autophagy rates were evaluated. MRI imaging revealed that average tumor size in the LP group (495.8 ± 22.3 mm^2^) was smaller than the control groups (810.3 ± 56.4 mm^2^; *P*<0.05). The mean survival time in the LP group (22.2 ± 2.1 d) was longer than control animals (16.0 ± 1.8 d; *P*<0.05). LP resveratrol-treated glioblastomas showed less Cyclin D1 staining, enhanced autophagy with up-regulated LC3 and Beclin1 expression, and widely distributed apoptotic foci around tumor capillaries with suppressed STAT3 expression and nuclear translocation. In conclusion, LP-delivered resveratrol efficiently inhibited orthotopic rat glioblastoma growth by inactivating STAT3 signaling and enhancing autophagy and apoptosis.

## INTRODUCTION

Glioblastoma multiforme (GBM) is the most common primary brain malignancy with extremely poor prognosis [1]. Surgery is the first stage of treatment of GBMs, while it can at most remove 99% of GBM mass, leaving 1% of the tumor cells for adjuvant chemo- and/or radio-therapy [2]. However, the susceptibility of normal brain tissue to radiotherapy [3] and the frequent resistance of glioblastoma cells to conventional anticancer drugs such as temozolomide (TMZ) are the main therapeutic challenges [4, 5]. Consequently, only 3% of GBM patients with standard-of-care post-operative radiation and chemotherapy can survive over 5 years and the others died of recurrence within 15 months [6]. It is therefore in urgent needs to explore more effective, lesser toxic and the blood–brain barrier/BBB-permeable anticancer agents in the management of glioblastomas [7].

Accumulating *in vitro* data reveal that resveratrol exerts inhibitory effects on human brain malignancies including medulloblastoma [7, 8] and glioblastoma cells [9]. More importantly, resveratrol is able to cross blood brain barrier [10] and has little toxic effect on normal brain cells [11], suggesting its potential neurotherapeutic values in the treatments of GMBs and other neurological diseases. Nevertheless, resveratrol is quickly metabolized *in vivo* upon absorption, resulting in very low bioavailability and limited biological effects in the main organs, especially in the brain [12]. Due to these reasons, no datum has been so far available concerning the therapeutic efficacy of resveratrol for intracranial malignancies. Alternatively, it would be necessary to explore an administration approach by which the bioavailability of resveratrol in the brain can be increased to an effective anti-glioblastoma level.

Lumbar puncture/LP is one of the main administration routes for treating brain malignancies. By this approach, the drug concentration in the brain can reach to a high level with limited dose, within a very short time and without affecting other tissues and organs. To overcome the neurotherapeutic dilemma of resveratrol, we checked the diffusion efficiencies and bioavailabilities of LP-administered resveratrol in rat brains and found that high drug availability in the brain (65.662 ± 6.259 μM) could be achieved in this drug delivery manner [13]. Since the concentration of LP-administered resveratrol is 3 times higher than the intracellular concentration in rat RG2 glioblastoma cells treated by the effective dose (150 μM) of resveratrol, it would be possible that lumbar punctured resveratrol might exert inhibitory effects on the intracranial tumors. The current study thus aims to address this issue using a rat orthotopic glioblastoma model formed by resveratrol-sensitive RG2 cells.

## RESULTS

### Establishment of orthotopic glioblastoma model

**Figure 1:**
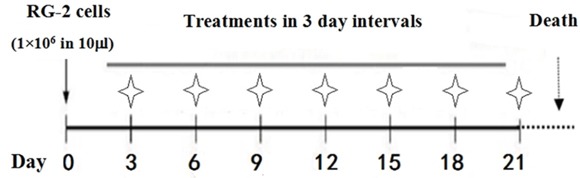
Flow diagram of lumbar puncture and drug administration schedule Treatments began 3 days after cell implantation (asterisks) via lumbar puncture with 100 μl of 300 μM (6.84 μg) resveratrol or 0.3% DMSO in 2-day intervals until death.

MRI scanning was performed on the rats after 3 days of RG2 cell intracranial injection. The images revealed slightly low signal changes in the left caudate nucleus region. After enhancing the images with Gd-DTPA, small solid tumors could be clearly observed in the brains of all rats with intracranial RG2 cell inoculation. The tumor-bearing rats were randomly separated to the Group 1, LP of 100 μl 0.3% DMSO-containing normal saline and Group 2, LP of 100 μl 300 μM resveratrol. The treatments were conducted according to drug flow diagram of lumbar puncture and administration schedule (Figure 1).

### Sufficient resveratrol availability in brain and tumor tissues

**Figure 2:**
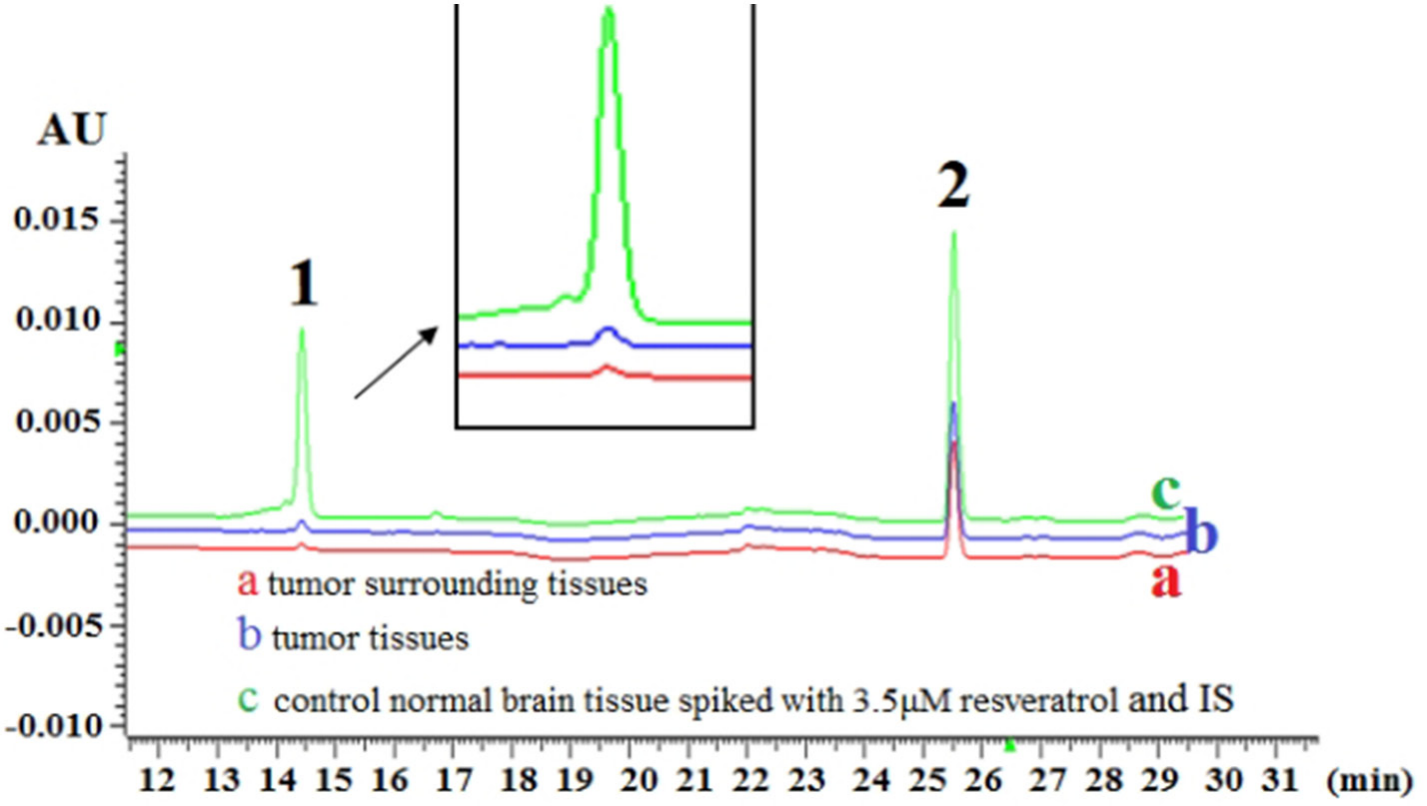
Trans-resveratrol concentrations in 0.08 g of glioblastoma and surrounding brain tissues 20 min after lumbar puncture with 100 μl 300 μM resveratrol HPLC/DAD chromatogram of (a) surrounding tissue, (b) tumor tissue and (c) control normal brain tissue spiked with 3.5 μm resveratrol and internal standard (IS). Peaks: 1. trans-resveratrol; 2. 1, 8-dihydroxy anthraquinone (IS).

The amounts of resveratrol in 0.08 g of rat intracranial glioblastomas and tumor-surrounding brain tissues were counted at 20 min after LP administration of trans-resveratrol. As shown in Figure 2, the average resveratrol concentration was 1.392 ± 0.281 nmol/g in tumor tissues and 1.037 ± 0.203 nmol/g in the tumor-surrounding ones. HPLC analysis revealed that only one peak corresponding to trans-resveratrol could be detected in the tumor extracts.

### Resveratrol inhibited tumor growth

**Figure 3:**
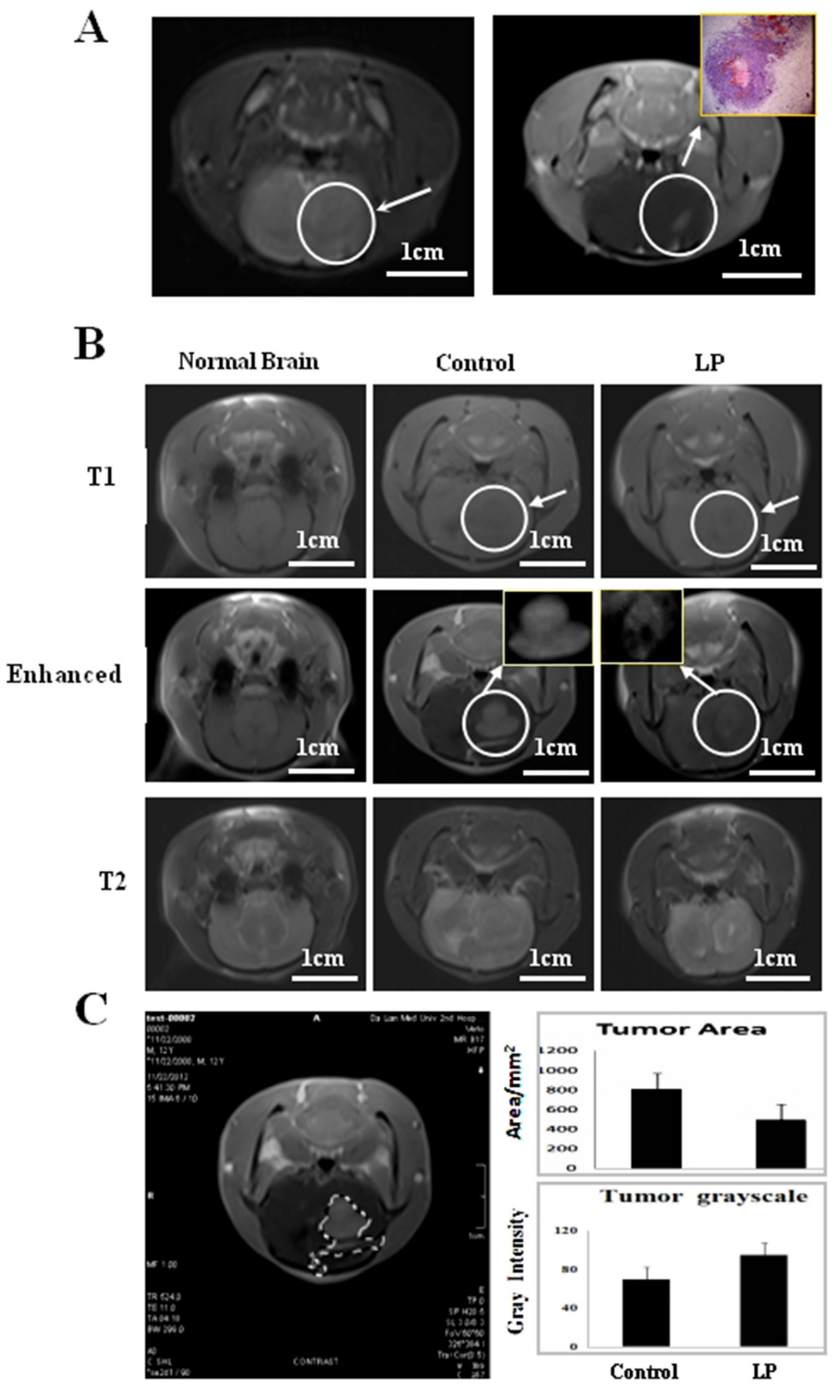
Monitoring tumor growth by magnetic resonance imaging (MRI) **A.** Left side: Conventional MRI imaging at Day 3 after transplantation, the arrow indicates the inoculation region. Right side: Enhanced imaging and the corresponding pathological finding (Inset) at Day 3 after intracranial transplantation. **B.** MRI 9 days after drug treatment. The arrow indicates the tumor location with higher magnification in the insets. **C.** Tumor areas were measured using Adobe Photoshop CS4. Average tumor area and gray intensity were determined according to area calculation performed on the tumor marked out from the MRI of the two experimental groups.

MRI imaging (Figure 3A) and histological examination (inset of Figure 3A) revealed solid tumor formation 3 d after RG2 inoculation. Treatment commenced on Day 3 and was performed in 2-day intervals until animals were sacrificed according to the study protocol. MRI was employed to monitor tumor growth and revealed that tumors occupied a great fraction of the left hemisphere, pressing the brain middle fissure in the control rats on Day 12 and in resveratrol-treated rats on Day 17. The force of the solid tumor pressing the brain middle fissure was more distinct in the control animals (Figure 3B). MRI monitoring showed that average tumor size in the resveratrol-treated group (495.8 ± 22.3 mm^2^) was smaller than the control group (810.3 ± 56.4 mm^2^; P<0.05). As shown in Figure 3C, extensive cell death with high overall gray intensity (94.7 ± 3.8) was observed inside the resveratrol-treated tumor tissues compared with the control group (69.6 ± 9.4).

### Resveratrol prolonged tumor-bearing times

**Figure 4:**
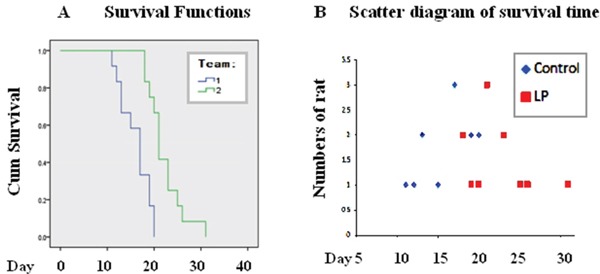
Prolonged survival of tumor-bearing rats treated with lumbar puncture-delivered resveratrol **A.** Survival curves of the tumor-bearing rats treated with lumbar puncture-administered resveratrol. Group 1, Control; animal number: 12; Group 2, intermittent lumbar puncture; animal number: 12. **B.** Scatter diagram of the survival times. Control: 12 tumor-bearing animals without treatment; LP: 12 tumor-bearing animals treated with resveratrol.

To further evaluate the therapeutic values of lumbar punctured resveratrol in treating rat orthotopic glioblastomas, the tumor bearing spans of the two experimental groups were estimated. As shown in Figure 4A and 4B, the control tumor-bearing animals had a mean survival time of 16.0 ± 1.8 days, while the mean survival time of the rats treated by lumbar punctured resveratrol was prolonged to 22.2 ± 2.1 days. Statistical analyses confirm distinct differences of the animal survival times and survival rates between the two experimental groups (P<0.05).

### Unique location of apoptotic foci in resveratrol-treated tumor tissues

**Figure 5:**
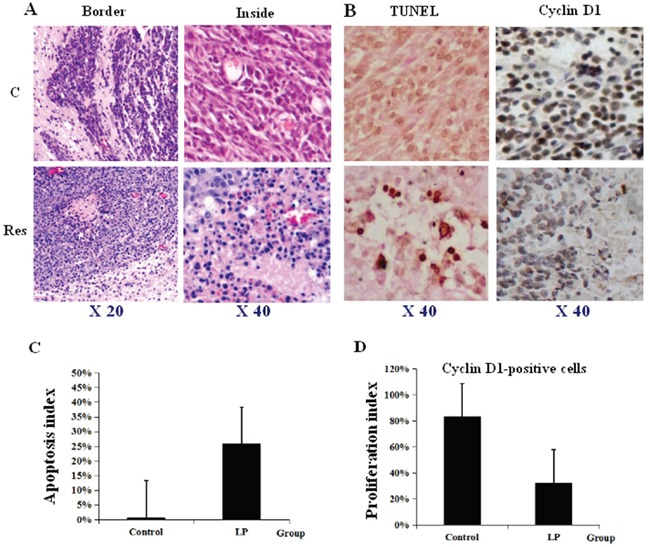
Lumbar puncture-administered resveratrol inhibits proliferation and enhances apoptosis and autophagy in orthotopic rat glioblastomas **A.** Illustration of less aggressive growth (Border) and distinct cell death (Inside) caused by lumbar puncture (LP) resveratrol through HE staining. **B.** TUNEL labeling and Cyclin D1 immunohistochemical staining performed on the tumors treated by lumbar punctured 0.3% DMSO (C) and resveratrol (Res). **C, D.** The incidences of TUNEL-positive and Cyclin D1-positive cells in the two experimental groups.

Because resveratrol suppresses the *in vitro* growth of RG2 glioblastoma cells and causes apoptosis [14], its corresponding effects on RG2 orthotopic tumors were evaluated. Hematoxylin and eosin (HE) staining demonstrated extensive cell death (Figure 5A). TUNEL assay revealed that apoptotic foci were widely distributed in the tumor tissues, especially at regions surrounding capillaries (Figure 5B and 5C; P<0.05). In contrast, cell death was uncommon in tumors from the control group. Immunohistochemical staining showed decreased frequencies of Cyclin D1 labeling cells in tumors treated with resveratrol (Figure 5B and 5C; P<0.05).

### Resveratrol enhanced autophagy

**Figure 6:**
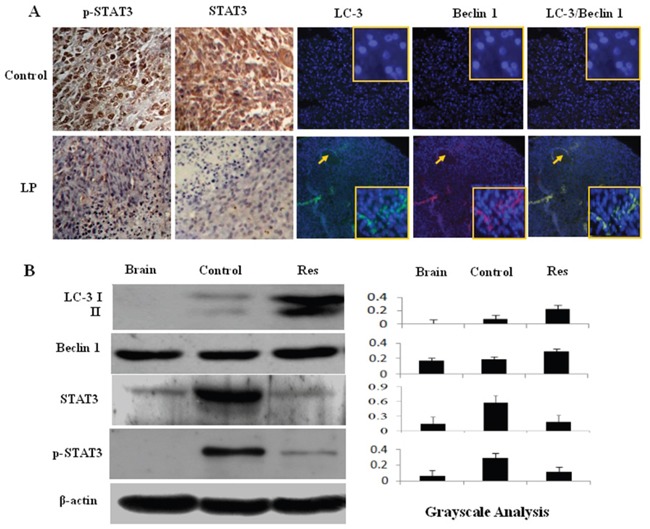
Effects of lumbar puncture-administered resveratrol on STAT3 signaling and autophagy-related protein expression **A.** p-STAT3 immunohistochemical staining and immunofluorescence (IF) co-labeling of LC3 (green) and Beclin1 (red) were performed on rat intracranial glioblastomas treated by lumbar puncture with 0.3% DMSO (Control) or trans-resveratrol (Res); the insets are the enlarged images (×40). **B.** Western blot analysis of LC3, Beclin1, STAT3 and p-STAT3 expression in normal brain (Brain) and RG2-derived orthotopic tumors treated by lumbar puncture with 0.3% DMSO (Control) or resveratrol (Res). β-actin was used as quantitative control for calculating LC3, Beclin1, STAT3 and p-STAT3 levels in densitometry analyses.

Increased autophagy reflects the stress-response status of drug-treated cells, although its precise biological consequence has not yet been ascertained [15]. The results of double immunofluorescence staining demonstrated that the autophagy-related protein LC3 was undetectable in glioblastoma tissues from the control group but was distinct in resveratrol-treated tumor tissues; Beclin1 was expressed in control tumor tissues and upregulated following resveratrol treatment (Figure 6A). The results of western blotting were in accordance with the immunofluorescence findings, showing elevated Beclin1 and the appearance of LC3 expression with remarkable LC3 II generation in the tumors treated with LP resveratrol (Figure 6B; P<0.05). Beclin1 was expressed but LC3 was undetectable in normal brain tissue.

### Resveratrol inactivated STAT3 signaling

ImmunohistochemistryforSTAT3 was performed on tissue microarrays constructed with the brain tumors and surrounding tissues of the animals with and without resveratrol treatment. Staining showed that STAT3 and p-STAT3 were undetectable in normal brain tissues, expressed at a high level in untreated glioblastoma tissues and remarkably decreased in resveratrol-treated tumors, particularly in the regions with extensive cell death (Figure 6A). Western blotting (Figure 6B) further demonstrated the reduction of STAT3 and p-STAT3 levels in tumors treated with LP resveratrol (P<0.05).

## DISCUSSION

The main challenges in glioblastoma treatment are the severe side effects of commonly used anticancer drugs and primary and secondary drug resistance [16, 17]. Trans-resveratrol possesses anti-glioblastoma activity without affecting normal brain cells [12, 18, 19] and LP administration can remarkably increase intracranial resveratrol concentrations [13]. However, it is still unclear whether the growth of intracranial tumors can be controlled and the survival times of glioblastoma-bearing rats can be prolonged by LP-delivered resveratrol. To address these issues, an orthotopic rat glioblastoma model was established using RG2 glioblastoma cells [20]. MRI images showed that although the intracranial tumors grew irrespective of LP resveratrol treatment, the speed of tumor growth and average tumor size were reduced in resveratrol-treated rats, which significantly prolonged mean survival time. The observed inhibition of tumor growth and the prolonged survival times result from LP-delivered resveratrol rather than the cytotoxic effects of 0.3% DMSO because 1) control rats were treated with the same concentration of DMSO (0.3%) in normal saline via lumbar puncture and 2) the amount of resveratrol in the brain tumor tissues was higher than in resveratrol-suppressed RG2 cells [13]. Nevertheless, tumor-bearing rats receiving LP resveratrol treatments eventually died from tumor expansion, suggesting the insufficiency of this therapeutic approach by itself to cure orthotopic glioblastomas. In clinical settings, surgery is the first-line treatment for glioblastomas [21], while chemotherapy is used pre-operatively to improve resection rates [22] and/or post-operatively to prevent recurrence [23, 24]. In this context, a combination of LP-administered resveratrol with surgery may improve the therapeutic outcome of the individual treatments, and we are currently testing this hypothesis using the same experimental model.

Resveratrol manifests its anticancer effects by suppressing proliferation and causing extensive cell death [20]. However, corroborating data from *in vivo* systems have not yet been available. Our morphological examination revealed that untreated tumor tissues showed aggressive growth and abundant Cyclin D1 expression, while TUNEL+ apoptosis foci were commonly observed in resveratrol-treated tumor tissues, which also showed a remarkable reduction of Cyclin D1 expression. The heterogeneous gray intensity inside the MRI-imaged resveratrol-treated tumors further supports these findings. Interestingly, those foci were commonly located in the regions close to or surrounding capillaries. Glioblastomas are highly vascularized [25], which may increase the diffusion efficiency of LP resveratrol from tumor capillaries, allowing it to directly act on neighboring tissues. Meanwhile, the brain-associated UDP-glucuronosyltransferases 1A6, 2B7 and 8, which metabolize trans-resveratrol to the less potent monoglucuronide are down-regulated in RG2 cells [14]. Consequently, the enhanced angiogenesis and reduced bio-transforming activities may increase the bioavailability and anticancer effects of trans-resveratrol in RG2-derived tumors. The above findings provide direct evidence for the effectiveness of LP resveratrol in inhibiting orthotopic glioblastoma growth.

Autophagy rates reflect cellular responses to environmental stresses such as drug treatment [26, 27]. So far, no report has been available concerning the status of autophagy and its relevance to resveratrol sensitivity in glioblastoma cells either *in vitro* or *in vivo*. We found that resveratrol promoted expression of the autophagy-related protein Beclin1 and, especially, LC3 in tumor tissues. Interestingly, the cells with Beclin1 and LC3 co-labeling also accumulated in regions close to capillaries, overlapping with the regions of high apoptosis. This finding indicates a potential link between enhanced autophagy and apoptotic cell death and suggests that these two biological events underlie the *in vivo* effects of resveratrol on glioblastomas.

STAT3 signaling is critical for glioblastoma cells [28] and is the major molecular target of resveratrol [29]. Because STAT3 signaling is activated in RG2 cells, its status in resveratrol-treated tumors was also evaluated. The results revealed that activated STAT3 signaling was suppressed by resveratrol; overall STAT-3 expression was down-regulated as was p-STAT3 nuclear translocation. Interestingly, STAT3 signaling was unevenly inactivated in the tumor tissues, and regions showing STAT3 inhibition exhibited extensive cell death. The roles of STAT3 signaling in suppressing apoptosis and autophagy in cancer cells have been well documented [30, 31]. For this reason, the cell death in those regions may be, at least in part, the biological consequence of resveratrol-mediated STAT3 inactivation.

Taken together, our imaging, pathological and mechanistical examinations confirm the inhibitory effects of LP-administered resveratrol on orthotopic rat glioblastomas, indicating the potential values of this therapeutic approach in the clinical treatment of brain malignancies. The sporadic distribution of STAT3-inactivated apoptotic foci in tumor tissues and the eventual death of resveratrol-treated tumor-bearing animals suggest the necessity to combine LP resveratrol with surgical resection for more promising therapeutic outcomes. Employment of improved drug delivery systems such as biodegradable nanocapsules [32] may further enhance the efficacy of resveratrol.

## MATERIALS AND METHODS

### Resveratrol delivery via lumbar puncture

A 100-mM stock solution of resveratrol (Sigma-Aldrich, St. Louis, MO, USA) was made by dissolving 0.228 g in 10 ml dimethyl sulfoxide (DMSO; Sigma-Aldrich). The 300 μM working solution was prepared by mixing 3 μl of the stock solution with 1 ml physiological saline just before use. The tumor-bearing rats were randomly separated into two groups: Group 1 received LP with 100 μl of 0.3% DMSO-containing normal saline (12 rats) and Group 2 received LP with 100 μl of 300 μM resveratrol (12 rats). Because the total cerebrospinal fluid (CSF) volume present in the rat central nervous system is about 500 μl [34], the final resveratrol concentration in the CSF was approximately 50 μM. LP was conducted into the L5-6 interspace in two-day intervals using previously described methods [13] and lasted until animal death (Figure 1).

### MRI monitoring of tumor growth

Animals were imaged at the MRI Facility of the Department of Radiology, the Second Clinical College, Dalian Medical University, using a 7-Tesla/30 cm horizontal bore magnet (SIEMENS Verio 3.0 T, Munich, Germany) 3 days after RG2 cell inoculation. Animals were anesthetized with 10% chloral hydrate (0.3 ml/100 g) by intraperitoneal injection, and then placed in an MR probe head in the prone position. A head surface coil was used for receiving the induced MR signal. A quadrature volume coil (72-mm inner diameter) was used to transmit radiofrequency pulses (Bruker BioSpin MRI GmbH). Check Sequence: T1WI, T2WI; Specific imaging parameters are as follows: whole brain axial scans. T1WI: SE sequence, layer thickness: 3 mm, interlayer spacing: 0.3 mm, FOV: 60 × 60 mm, matrix: 326 × 384, TR/TE = 524 ms/11 ms, imaging time: 212 s; T2WI: TSE sequence, layer thickness: 3 mm, interlayer spacing: 0.3 mm, FOV: 60 × 60 mm, matrix: 288 × 384, 2NEX TR/TE = 4000 ms/81 ms, imaging time: 116 s; DWI: TSE sequence, layer thickness: 3 mm, interlayer spacing: 0.3 mm, FOV; 144 × 144 mm, matrix: 130 × 130, 8NEX TR/TE = 7082 ms/90 ms, imaging time: 249 s. The contrast agent (Gd-DTPA, 0.3 mmol/kg) was injected through tail veins for enhanced imaging [3, 35].

### Tumor area measurements

For accurate tumor area measurements, digital MRI images of the intracranial tumors were saved in JPG form and opened at Adobe Photoshop CS4 site (Adobe Systems Incorporated, San Jose, CA, USA). As shown in Figure 3C, tumor margins were defined using the “Magic Wand” tool. Pixel values from individual tumors were calculated using previously described methods [36]. A 1 mm^2^ standard area unit was set in the original films/MRI images and its pixel value was defined as the numerator. The areas of all tumors were calculated simultaneously by dividing each of the tumor pixel values (Y) in the excel form with the pixel value of standard area unit (X) and then multiplied by the real area of the standard unit (Z). Calculations were conducted by three independent researchers, and the data obtained were analyzed together by the independent-samples t-test method with SSPS 17.0 (SPSS Inc., Chicago, IL, USA). Statistical significance was defined as P<0.05.

### Sample collection and treatments

Tumor-bearing animals that suffered from rapid weight-loss, lack of appetite and/or paralysis were painlessly sacrificed in a cold room (4°C) by an authorized expert in the DMU Animal Center through cervical dislocation, and whole brains were harvested within 3 minutes. All animal experiments were performed under chloral hydrate anesthesia and all efforts were made to minimize animal suffering. By the end of the experiments, the animals were scarified by the experts in the University Animal Center. Portions of the tumor-containing tissues were snap frozen with evaporated liquid nitrogen (−80°C) for frozen sectioning and protein preparation, and the remaining tissue was fixed in 10% formalin, embedded into paraffin and sectioned for morphological and immunohistochemical examinations using standard methods [37].

### Resveratrol availability in brain

All experiments were performed 20 min following LP administration. Briefly, brains were dissected on an ice bed, wrapped in aluminum foil, snap frozen in liquid nitrogen and stored at −80°C until use. Tumor-bearing and cancer-free brain tissues in the frozen sections were separately collected using standard procedures [13] and used for HPLC analysis. Sample tissues were extracted with 416 μl methanol and 84 μl internal standard (IS, 1, 8-dihydroxyanthraquinone, 200 μg/ml) in a centrifuge tube. The tissue homogenates were vortexed for 5 min and centrifuged at 12000 rpm for 10 min at 4°C. The supernatant was then transferred to a clean tube. The residue was extracted twice more with 1 ml methanol by vigorous 5 min agitation, followed by centrifugation. The combined organic solvent of the supernatants was evaporated to a final volume of 400 μl, and subsequently placed in a sealed amber vial for HPLC analysis [11]. To improve the sensitivity and precision of quantification, we purified the samples and cited 1, 8-dihydroxyanthraquinone (Sigma-Aldrich) as the internal standard and trans-resveratrol (Sigma-Aldrich) as the standard for drawing a standard curve using previously described methods [13].

### Cellular and molecular responses of tumor cells to resveratrol

Paraffin-embedded xenografts were sliced into 7 μm sections, which subjected to HE staining. In parallel, immunohistochemical staining was performed adjacent sections using a mouse anti-Cyclin D1 antibody (1:80; DAKO, Glostrup, Denmark) to evaluate tumor proliferation [38]. Terminal deoxynucleotide transferase (TdT)–mediated dUTP-biotin nick-end labeling (TUNEL) assay was conducted to detect apoptotic cells in the tumor tissues according to manufacturer's instructions (Promega, Madison, WI, USA) [39]. Because STAT3 signaling is the main molecular target of resveratrol and the critical survival and growth-promoting factor of glioblastoma cells [40, 41], its status in the orthotopic glioblastoma tissues with and without LP resveratrol treatment was analyzed by immunohistochemical staining and western blotting using rabbit anti-STAT3 antibody (1:300 for immunohistochemistry and 1:1000 for western blotting; Santa Cruz Biotechnology, Santa Cruz, CA, USA) and anti-phosphorylated STAT3 antibody (1:300 for immunohistochemistry and 1:600 for western blotting; ProteinTech, Chicago, IL, USA) [41]. Binding of the primary antibody was detected by a peroxidase reaction using 3,3-diaminobenzidine tetrahydrochloride (DAB) as the substrate (Vector Laboratories, Burlingame, CA, USA). Tissue sections without primary antibody incubation were used as background controls for IHC staining and β-actin was used as a quantitative control for western blotting.

### LC3 and Beclin1 double immunofluorescence labeling

LC3 and Beclin1 double immunofluorescence labeling was performed on tumor tissues obtained from the two experimental groups. The tumor tissues were rinsed with PBS, and then fixed in cold acetone for 20 min and stored at −20°C. After being blocked with 10% goat serum in PBS for 20 min, tumor tissues were incubated overnight at 4°C with mouse anti-LC3 antibody (1:80; GeneTex Inc., Irvine, CA, USA) and rabbit anti-Beclin1 antibody (1:100; Abcam, Cambridge, UK), followed by co-incubation with FITC-conjugated goat anti-mouse IgG and PE-conjugated goat anti-rabbit IgG (both 1:100; Santa Cruz Biotechnology) at 37°C for 60 min in the dark. Nuclei were labeled with 4,6-diamidino-2-phenylindole, 2-(4-amidinophenyl)-1H-indole-6-carboxamidine. After being sealed with fluorescence mounting medium, the tumor tissues were observed and imaged under a fluorescence microscope (BX53F, Olympus, Tokyo, Japan). The same anti-LC3 and anti-Beclin1 antibodies were used for western blotting at dilutions of 1:200 and 1:600, respectively.
